# Low Velocity Impact Behavior of Glass Filled Fiber-Reinforced Thermoplastic Engine Components

**DOI:** 10.3390/ma3042463

**Published:** 2010-03-31

**Authors:** Zakaria Mouti, Keith Westwood, Kambiz Kayvantash, James Njuguna

**Affiliations:** 1 Centre for Automotive Technology, Department of Sustainable Systems, Cranfield University, Bedfordshire, MK43 0AL, UK; E-Mails: z.mouti@cranfield.ac.uk (Z.M.); k.kayvantash@cranfield.ac.uk (K.K.); 2 Eaton, Automotive Group, West Midlands, DY5 2LB, UK; E-Mail: keithwestwood@eaton.com (K.W.)

**Keywords:** oil pan, sump, impact resistance, polyamide, glass fibre-reinforced composite, thermoplastic

## Abstract

This paper concerns automotive parts located underneath the engine and in particular the engine oil pan. Classically made of stamped steel or cast aluminum, new developments have allowed the manufacture oil pans with polyamide 66 reinforced by 35% weight of short glass fiber. However, polyamides have some limitations and the most significant is their response to localized impact loading. The nature of the impact considered here is of a typical stone collected from the road and projected into the oil pan.  Low velocity impact investigations were carried out using a gas gun and drop weight tower.  The study shows that the design of the oil pan has a significant contribution in the shock absorption. In addition to the material properties, the geometry and the ribbing both cleverly combined, increase the impact resistance of the component significantly. Areas of oil pan design improvement have been identified and conclusions drawn.

## 1. Introduction

A main concern in the automotive industry is vehicle weight reduction so as to help reduce fuel consumption and therefore emission levels. One way to realize this objective and meet the challenge of cost and performance is by the use of glass-reinforced thermoplastics composites. They offer distinct advantages over more conventional engineering materials such as aluminum and steel including higher specific strength and stiffness and superior corrosion resistance as well as improved fatigue properties. Thermoplastic composites used in automotive applications can be short fiber-reinforced thermoplastic, or long fiber-reinforced thermoplastic composites. Injection molded short fiber-reinforced thermoplastic composites are currently the most prevalent thermoplastic composites.

There are many studies that have been performed in order to investigate the impact properties of thermoplastic composites [1,2,3,4,5,6,7,8,9,10]. Low velocity impacts are known to induce damage to the composite in the form of matrix cracking, delamination, debonding and fiber breakage. A number of studies on the low-velocity impact performance of thermoplastic-matrix composites have been conducted but, in most cases, the composites were fully laminated into relatively rigid plates made of poly(ethylene terephthalate) [2,6], polypropylene [4,8,9], polyethylene [10,11,12,13] and PEEK [14,15] fiber reinforcement. Research has shown that composites are capable of absorbing energy and dissipating it by various fracture and elastic processes when subjected to a low velocity impact [8]. The ability of these materials to absorb energy elastically depends on the mechanical properties of the matrix and fibers, the interfacial strength, the velocity of impact and the size of the component. Polymer matrix composites are known to be highly susceptible to internal damage caused by transverse loads even under low velocity impact [9]. For the effective use of polymer matrix composites in higher performance applications, it is important to understand the cause of damage formation under low velocity impact conditions as well as the potential for improvement of damage resistance characteristics of composites.

Despite increased use in under-the-hood applications, published work on the impact behavior of polyamide short fiber-reinforced thermoplastic composites is scarce. The polyamide (PA) family consists of different grades depending upon the way they were polymerized. PA6 and PA66 are the most popular forms of polyamides [16]. More than 40% of the PA6 and PA66 produced are consumed by the automotive market, principally for new under-the-hood applications. These materials have a particular utility in performing mechanical duties that traditionally relied on metal parts. However, polyamides have some limitations and the most significant of these is their response to localized impact loading. Thus, material substitution involves making sure that the new parts are service lifetime capable. Few available studies have looked into the fracture toughness and the impact behavior of polyamide plates’ samples but mainly focus on fiber content and length effects [17,18]. These studies found that the addition of fibers up to 35% weight of a polyamide matrix led to an improvement of fracture toughness with a minor advantage for long fibers. The impact resistance increased with the thickness; nonetheless, the relationship established was valid only in the examined range. Most impact studies use drop weight testing machine to assess the impact resistance of composites, but the impactor type and geometry used in reported studies are different and tests are mostly done on square plates, eliminating the geometry effect that a full component could have.

The concern of this paper is those components located underneath the engine and, in particular, the engine oil pan of light utility vehicles. Classically made of stamped steel or cast aluminum, a new way is explored to manufacture oil pans with polyamide 66 reinforced by 35% weight of short glass fiber. The nature of the impact we consider here represents typically a stone collected from the road and projected into the oil pan. The oil pan design is made in such a way that the connection to the engine is consistent with the metal construction previously used. A noticeable facet of this new design is the ribbing which gives a dimensional stability to the structure and also helps to keep the main structure safe after an impact by dissipating the impact energy. The impact resistance of a component is not only influenced by the material the manufacturing processes and the external conditions but also by the geometrical design employed. The ribs are allowed to break but no damage such as a crack or a hole should be visible on the component. Every upshot that could lead to a possible leak is to be avoided. The damage assessment is mainly realized by visual inspection and the material behavior under impact in micro-scale is not considered but only its overall contribution to impact resistance. Separate investigations are currently underway investigating the consequences of micro-fractures and are therefore currently beyond the scope of this article.

The oil pan impact test rig is designed to simulate the loading conditions to which the composite component is subjected whilst in operational service and hence failure modes and mechanisms likely to occur are reproduced. Our oil pan prototypes were tested under low velocity impacts with impact energies varying from 3-12 Joules depending on the impact locations. The most exposed areas have to undergo a maximum impact energy of 12 Joules which reflects the highest energy that the oil pan is likely to face from road debris/stones during normal road operations. We appreciate that material properties of the oil pan will evolve during the product life cycle because of working conditions and ageing effects. In this respect, many parameters involved are considered in this initial study while others have been fixed to reduce the complexity of the problem. The oil pan is impacted empty in neutral conditions at room temperature of 20 °C without oil. It has a unique design and material composition with no welded parts on it. The selected points of impact are located all around the oil pan. All the impacts are perpendicular to the target surfaces which have more critical effects than an angular impact that can rebound without having completely transferred its energy to the target. At this time, only a single impact scenario is presented; nevertheless multiple impacts scenario will be considered for further tests.

## 2. Materials and Experimental Techniques

### 2.1. Manufacture of the Oil Pans Prototypes

All the prototypes were supplied by Eaton Corp. (UK) and were manufactured using injection molding. This process is suitable to form complex shapes with excellent surface finish and good dimensional accuracy for high production rate and low production costs. The raw PA66-GF35 (PA66 with 35% weight glass fiber content) is melted and then injected into the mould, where it cools and solidifies into the final part. The process cycle consists of four stages: clamping, injection, cooling and ejection. Prior to the injection phase, the clamping unit pushes two mould halves together to keep the mould closed while the material is injected. Next, the raw plastic is melted by heat (265 to 295 °C) and pressure (around 100 bars), and advances towards the mould for fast injection while the build-up of pressure packs and holds the material. The molten plastic inside the mould begins to cool as soon as it makes contact with the interior mould surfaces into the desired shape. The packing of material allows additional material to flow into the mould to reduce the amount of visible shrinkage. After the required cooling time, the part is ejected from the mould. Following the cycle, some post processing is required. During cooling, the material in the mould’s channels solidifies while attached to the part. This excess material along with any flash that has occurred is trimmed from the part. For thermoplastics, the scrap material that results from this trimming is recycled. The oil pan formed weights around 2.4 kg. The oil pans overall dimensions are 600 × 300 × 150/70 mm. The main structure is 3-3.5 mm thick and the ribbing is approximately 2 mm thick and has variable ribs height.

Following the injection molding process, vibration welding is utilized for the integration of functional parts such as the oil deflector, to calm the flow of oil back to the sump, the oil pick-up pipe, the oil pump and so on. Those parts which cannot be molded directly during the injection process are welded to the oil pan afterwards using friction welding. The parts to be joined are vibrated, against each other at a specified frequency, amplitude and pressure which produces frictional heating of the surfaces, causing the polymer to melt at the interface. The molten polymer flows out of the weld-zone giving rise to flash. When the vibration stops, the weld cools and solidifies.

### 2.2. Stone Impact Phenomenon

**Figure 1:**
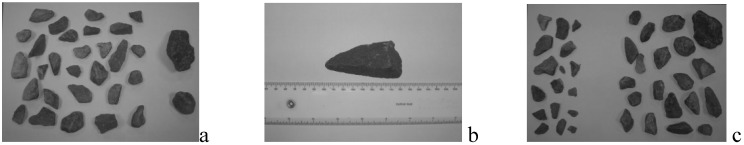
Selection of random stones collected on the road.

In order to quantify the size of stone likely to cause damage to an oil pan, a search and a selection of random granite stones are collected from regular roads (Figure 1). This approach is taken to allow measurement of the mass and size of road stones. The granite stones shown on the left of Figure 1a weigh less than 17 grams and all fit into the damaged area between two consecutive ribs. The two stones on right do not fit; the rounded stone weighs 21 grams and the larger stone weight 78 grams. There are variable gaps between the ribs in the area of impact. A 87 gram random stone with a triangular profile is able to fit into the gap where damage is evident (Figure 1b). A possible solution would be to reduce the rib spacing (Figure 1c) so as to exclude more stones.

**Figure 2:**
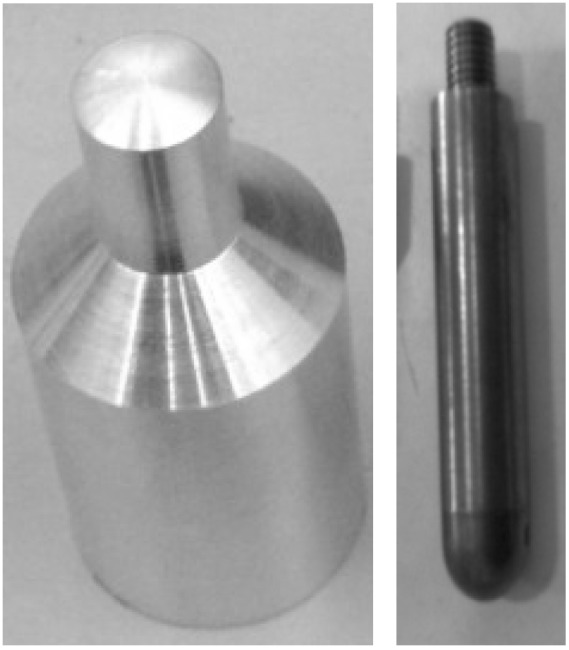
10 mm projectile (left) and 10 mm impactor (right).

In the oil pan prototypes, the maximum gap between the ribs is 13 mm. Therefore, this reduces both the mass and size of the projectile able to contact the base-molding wall but there is still a range of stones able to impact between the rib spacing which is the critical aspect. To simulate the effect of stones, an impactor and a projectile (Figure 2) were chosen according to the experiments selected. Both have a 10 mm diameter tip. The material used for the projectile is aluminum as it has nearly the same density as granite gravels (2700 kg/m^3^) whereas the impactor is made of steel. The testing parameters are defined in Table 1. The shape of the projectile is similar to the triangular stone shape and it also allows a more stable trajectory, and further allows the length of the pointed end to penetrate into structures as would happen with a typical stone shown in Figure 1(b).

**Table 1:** Stone impact phenomenon.

Variables	Attributes
Projectile	Shape, mass, density and material constants
Impact conditions	Velocity and angle of incidence
Ambient conditions	Temperature and humidity
Oil pan system	Type (material properties), thickness (injection molding), stiffness (ribs), high strain rate properties, moisture absorbed

### 2.3. Testing Equipment

All the low impact tests were conducted using a Rosand instrumented falling weight impact tester Type 5 and a low velocity air gun testing machine manufactured by Sabre Ballistics. The drop weight device was equipped with data acquisition system to acquire force versus time data. Impact energy and velocity can be varied by changing the mass and height of the dropping weight. The velocity of the falling drop mass is measured just before it strikes the specimen. It is also fitted with pneumatic rebound brake which prevents multiple impacts on the specimen. During the testing, the specimen is held in the fixture placed at the bottom of the drop tower which provided a clamped support span. The weight of the cross-head is maintained at a specific value and it is guided through two frictionless guide columns. The impactor end of the drop mass is fitted with an impact load sensor to record the transient response of the specimens. To carry out the impact tests, oil pans samples were placed between the clamps and the height was adjusted depending on the desired energy level. The impactor had a 10 mm diameter hemispherical tip. The transient force signal obtained during the test was measured using a piezoelectric load cell located above the impactor tip and was routed through an amplifier and logged against a time-base.

In the air gun, the desired projectile velocity is obtained by adjusting the pressure of the gas before firing. It obtains velocity by using a solenoid valve which releases a set volume of gas into a chamber within the gun. The 10 mm diameter projectiles are loaded by pushing forward the quick release barrel. The barrel is locked after loading and the gun is ready to fire. Firing is accomplished electrically and can be operated remotely and safely. A projectile velocity measuring system is mounted on the muzzle of the barrel and a camera records the impact event. A removable laser is mounted in the centre of the barrel allowing the alignment of the projectile with the desired target spot. The distance from the end of the barrel to the impact position is kept constant to 400 mm in order to allow reproducibility under identical impact conditions.

These two experimental methods (guided drop tower and projectile) were used. The drop tower provides more impact information but the impactor is constrained during the falling and has only one degree of freedom. The projectile method gives limited impact information but since it is a projectile, it is more representative of a stone impact.

## 3. Results and Discussion

### 3.1. Drop-Weight Testing

**Figure 3:**
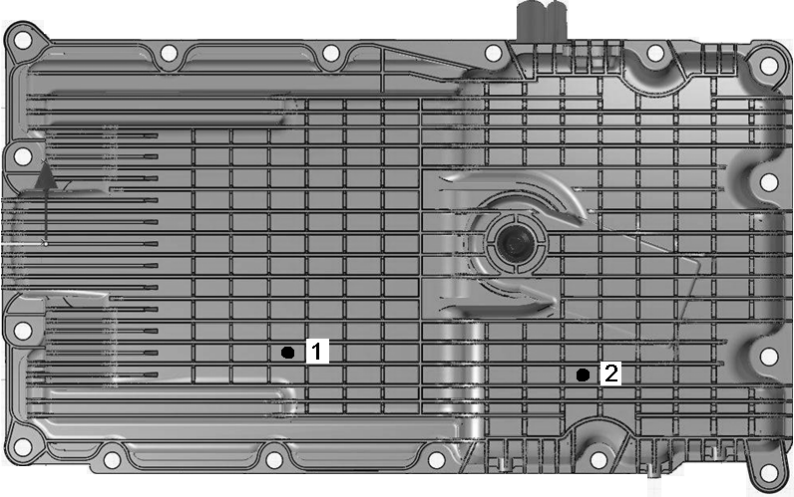
Two different impact locations highlighted.

The drop-weight tower allows accurate impacts on the desired target. Many impact positions were tested (over 1000 impacts taken) on different oil sumps and at different velocities. Figure 3 shows two locations that are representatives of all the impacts positions of the bottom oil pan.

**Figure 4:**
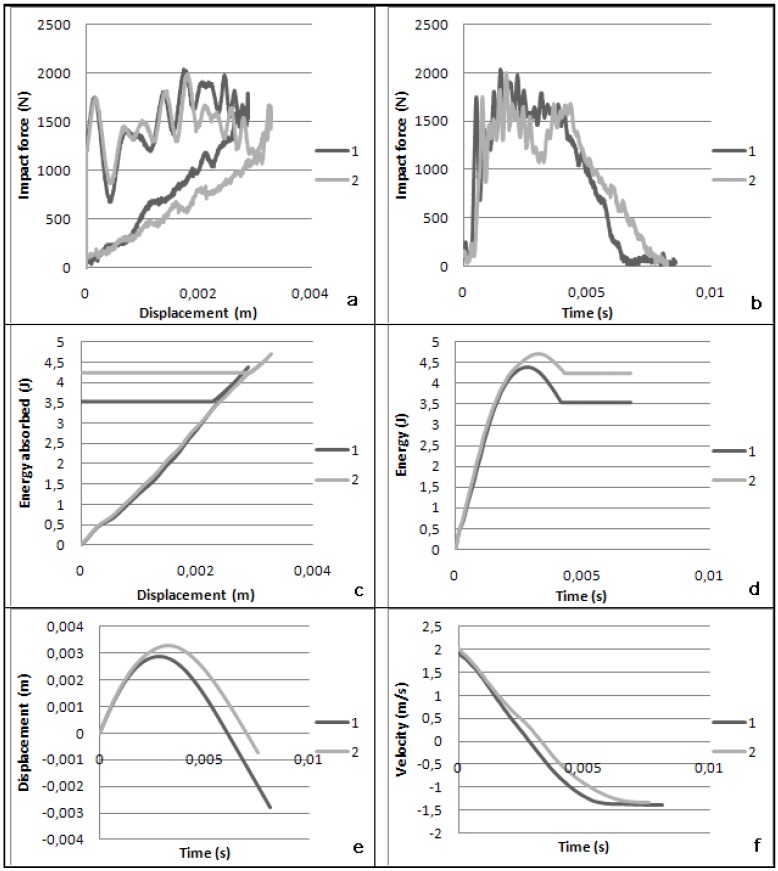
Data obtained after a 2.28 kg falling weight impact onto the oil pan at 5 Joules.

The magnitudes measured are force, displacement, energy, impact velocity and the duration time of the event. The forces histories in Figure 4a and Figure 4b show a large oscillations zone which can be an indication of material damage. When the force between the oil pan and the impactor returns to null, it is the end of contact time and the end of penetration. Figure 4a shows closed loops in which the area under the curve is the deformation energy that is initially transferred from the impactor to the oil pan surface and then given back from the oil pan surface to the rebounding impactor. The area included inside the loop refers to the energy absorbed during the impact. In the Figure 4c and Figure 4d, the energy grows until the maximum displacement is reached (3 mm) then the energy decreases until the impactor detaches from the oil pan surface. We consider that from this point to the end of the test, the oil pan surface does not dissipate energy any further, only releasing the residual part of the elastic energy stored during the penetration of the impactor. At the final instant, the energy value shown in the graph is equal to the dissipated energy. It is important also to notice that the maximum energy level is equal to the initial kinetic energy of the impactor. The maximum deflection for these tests was around 3 mm (Figure 4a and Figure 4c). They show that the component has recovered its initial position as deflections return to zero. The impact has reached its maximum penetration into the oil pan surface (Figure 4e) when the impactor velocity becomes zero (Figure 4f) [19].

According to the charts in Figure 4, it seems that the structure, and more specifically the impacted surfaces, have undergone the efforts in two different ways. There is a distinction to be made between impact 1 and impact 2. For impact 2, the forces histories demonstrate that the structure has undergone micro-damages if we look at the variations in force intensities before reaching the maximum force. The displacement histories confirm that the structure has recovered its initial position after oscillation and been displaced with the return to zero. The energy histories illustrate the energy absorbed by the component which is actually practically equal to the impact energy transferred by the impactor. Impact 2 has passed successfully the test as defined previously, however there was an oil pan rib broken which can be seen in the Figure 4b when the curve is falling down before beginning to increase again (at 3 ms). For impact 1, there is a small difference which could have an opposite outcome. The force history is quite similar to the other impact scenarios studied. Nevertheless, it is important to note that the return to zero force is quicker than the impact 2 and there is a small fall in the curve which could indicate a broken oil pan rib, as revealed in this case. The displacement history reveals that the structure has oscillated but it has not recovered after the impact and did not return to zero (not recorded in the chart). According to the energy history, it has not completely absorbed the impact energy from the projectile. Nonetheless, the pan has absorbed a big part of the impact energy and has released comparable energy as the other impact 2. This is actually difficult to interpret since a small detail could result in a big change in the component integrity and this component has a complex shape. The real observation has revealed the actual initiation of a crack zone.

### 3.2. Air Gun Testing

**Figure 5:**
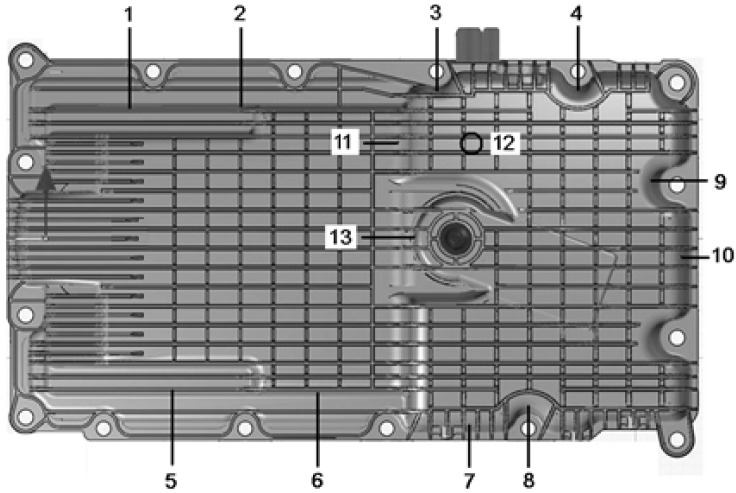
Map of the impact locations.

**Figure 6:**
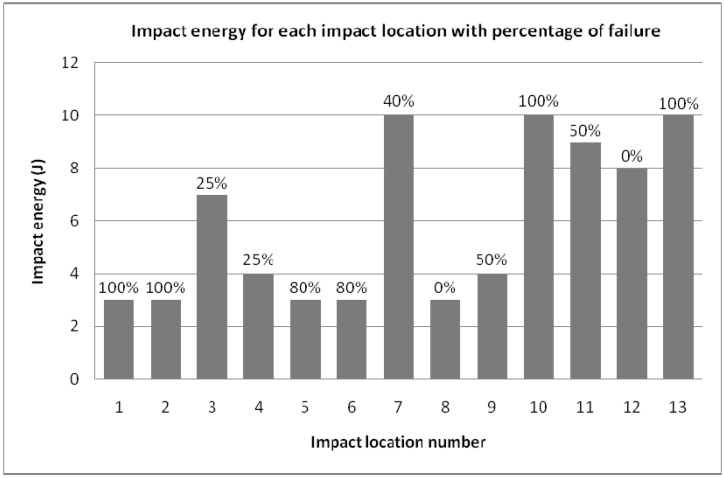
Percentage of failure for each impact location at different impact energies.

The 22 gram projectile was fired at the oil pan for a single impact perpendicular to the surface (Figure 5 and Figure 6). The impacts were reproduced at least four times using a new oil pan at each time.

Figure 5 shows some of the impacts realized on the oil pan. Depending on their location, the impact intensity used is variable. Figure 6 provides details of the impact energies applied for each impact location and the outcomes for each shot indicated in terms of percentage of failure in part integrity.

**Figure 7:**
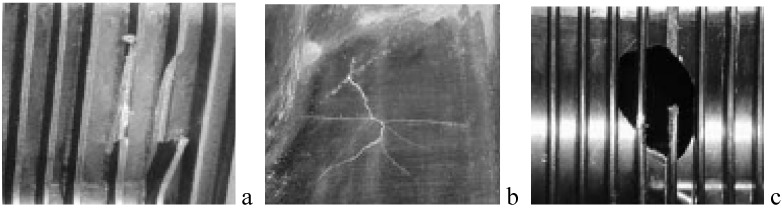
Damage types that can result after an impact. Broken ribs (a), Interior cracks (b), Perforation (c).

Figure 6 shows an overview of the impact limits that the oil pan can withstand and points out where the areas of weakness exist. The disparity in terms of impact resistance is clear. This is due to the complexity of the oil pan in its structure (with surfaces more or less stiffened in the inside of the oil pan) and its material arrangement (*i.e.,* fiber direction arrangement). The understanding of material failure is not easy. This judgment is blurred by the complexity of the material. Nevertheless, a resistance limit more or less constant was determined for each location shown in the map (Figure 5). Explicitly, the unlikely sides to receive an impact have to undergo 3-4 Joules, the edges of the pan and some medium level risk sides have to withstand 7-8 Joules. The most exposed areas should be able to withstand 10-12 Joules. The sides likely to fail in Figure 6 have to be improved, redesigned or stiffened. As expected, the ribbing is the relevant geometric parameter. It plays a big part in the impact resistance of the component. In addition, it is evident that the ribbing dimensions should be contained following the following values. The rib height should be between 4 to 10mm and for a height between 2-3 mm. For ribs less than these dimensions, it seems that the ribs are less efficient while above this height, the ribs are too fragile and brittle. Figure 7 shows various damage types that can result after an impact.

## 4. Concluding Remarks

Glass filled polyamides have improved mechanical properties but are processed at higher temperatures and absorb more moisture, which has a detrimental effect on impact performance. The overall stiffness of an oil pan is improved by the flow property of the resin that enables long flow distances, thus enabling reliable molding of thin-walled sections. Moreover, the stiffness and the strength of the component are also increased by adding ribs at the edge of the pan. The response to an impact loading and the way it dissipates the incident kinetic energy of flying road debris is far different to that of metals. For low and intermediate incident energies, metals absorb energy through elastic and plastic deformation. Although the latter may cause some permanent structural deformation, its consequences on the load-carrying capability of the component are usually small. At high incident impact energies, target perforation may occur and the passage of the impactor will generally result in petalling, cracking and spalling. Such damage will degrade the load-bearing ability of the structure; nevertheless, its effects can generally be predicted using fracture mechanics principles. In composites however, the ability to undergo plastic deformation is extremely limited with the result that energy is frequently absorbed in creating large areas of fracture with ensuing reductions in both strength and stiffness. Furthermore, the prediction of the post-impact load-bearing capability of a damaged composite structure is more difficult than for metals since the damage zone is generally complex in nature and consequently very difficult to characterize. An impact on a given target when its intensity is relatively close to the material failure limit, has variable outcome and therefore cannot be predicted accurately without considering a margin where the component could pass or fail the impact. From the original design of the oil pan, some weakness areas have been found. Most of them are surfaces without ribs being flattened surfaces mainly located on the sides of the oil pan. All the flattened surfaces potentially exposed to an impact have to be covered with ribs so as to stiffen the oil pan structure. Some areas already protected with ribs are still showing weakness points and must receive more ribs. An increase of rib density can be obtained by reducing the rib spacing in an attempt to decrease the flat surfaces exposed to an impact. Further research work is under way and will be reported in the future.
